# Announcement

**Published:** 2015-07-31

## Additional Guidance Online for Providers Regarding 9-Valent HPV Vaccine Use Among Persons Who Previously Received HPV Vaccination

A 9-valent human papillomavirus (HPV) vaccine (Gardasil 9, Merck and Co., Inc.) was licensed for use in females and males in the United States in December 2014 (1,2). This is the third HPV vaccine licensed by the Food and Drug Administration; the other vaccines are the bivalent HPV vaccine, licensed for use in females, and the quadrivalent HPV vaccine, licensed for use in females and males (3).

In February 2015, the Advisory Committee on Immunization Practices (ACIP) recommended 9-valent HPV vaccine as one of three HPV vaccines that can be used for routine vaccination of females and one of two HPV vaccines for routine vaccination of males. ACIP recommendations were published in a March 2015 report (4). Additional information has been posted on the CDC website to provide guidance on issues that were not addressed in the March report but are likely to arise during the transition to 9-valent HPV vaccine, including questions about use of 9-valent HPV vaccine among persons who previously received bivalent or quadrivalent HPV vaccine (http://www.cdc.gov/vaccines/who/teens/downloads/9vHPV-guidance.pdf).
